# Pneumonia Risk Associated with the Use of Individual Benzodiazepines and Benzodiazepine Related Drugs among the Elderly with Parkinson’s Disease

**DOI:** 10.3390/ijerph18179410

**Published:** 2021-09-06

**Authors:** Kuang-Hua Huang, Chih-Jaan Tai, Yu-Hsiang Kuan, Yu-Chia Chang, Tung-Han Tsai, Chien-Ying Lee

**Affiliations:** 1 Department of Health Services Administration, China Medical University, Taichung 40402, Taiwan; khhuang@mail.cmu.edu.tw (K.-H.H.); dondon0525@gmail.com (T.-H.T.); 2 School of Medicine, China Medical University, Taichung 40402, Taiwan; cjtai@mail.cmu.edu.tw; 3 Department of Otorhinolaryngology, China Medical University Hospital, Taichung 40402, Taiwan; 4 Department of Pharmacology, Chung Shan Medical University, Taichung 40201, Taiwan; kuanyh@csmu.edu.tw; 5 Department of Pharmacy, Chung Shan Medical University Hospital, Taichung 40201, Taiwan; 6 Department of Long Term Care, National Quemoy University, Kinmen 892009, Taiwan; iamcgaga@gmail.com; 7 Department of Healthcare Administration, Asia University, Taichung 41354, Taiwan; 8 Department of Medical Research, China Medical University Hospital, Taichung 40402, Taiwan

**Keywords:** benzodiazepines, benzodiazepine related drugs, pneumonia, Parkinson’s disease

## Abstract

Most patients with Parkinson’s disease (PD) gradually develop oropharyngeal dysphagia which is often associated with pneumonia risk. The possible association of benzodiazepine (BZD) and benzodiazepine related drugs (BZRD) use with pneumonia risk has received increasing attention but remains controversial. We investigated pneumonia risk associated with the use of BZDs and BZRDs in older adult patients with PD. This case-control study analyzed data of 551,975 older adult patients with PD between 2001 and 2018 in Taiwan. To minimize potential confounding, we used 1:4 propensity score matching to include older adult patients without pneumonia as controls. Incident pneumonia risk was significantly higher in current (adjusted odds ratio (aOR) = 1.25, 95% CI = 1.23–1.27) and past (aOR = 1.13, 95% CI = 1.11–1.15) users of BZDs. Regarding BZRDs, recent (aOR = 1.08, 95% CI = 1.06–1.11) and past (aOR = 0.89, 95% CI = 0.88–0.91) users had higher and lower risks of incident pneumonia, respectively. Pneumonia risk varied based on their use of BZDs and BZRDs. In these individuals, incident pneumonia risk was high in users of BZDs, such as midazolam, lorazepam, flunitrazepam, estazolam, and clonazepam. Regarding the use of BZRDs, zopiclone increased incident pneumonia risk.

## 1. Introduction

With Parkinson’s disease (PD) progression, the bulbar muscles get affected, leading to dysphagia. Dysphagia is a common symptom in patients with PD and may occur at any stage of the disease. Most patients with PD gradually develop oropharyngeal dysphagia [1]. Patients with oropharyngeal dysphagia have difficulty swallowing and are associated with increased aspiration pneumonia risk [2].

Sleep disturbance is a common nonmotor symptom among patients with PD, which may affect their quality of life. Sleep disorders can affect 38% to 98% of patients with PD [3]. These patients with sleep disturbance are commonly prescribed hypnotics [4]. Benzodiazepine receptor agonists (BZRAs), including benzodiazepines (BZDs) and benzodiazepine related drugs (BZRDs), act on the gamma-aminobutyric acid (GABA) type A receptor and are the mainstay treatments for insomnia [5]. PD is characterized by the progressive loss of dopaminergic neurons and neuronal degeneration of the substantia nigra [6]. Animal studies have shown that GABA agonists decrease extracellular striatal dopamine concentrations [7]. Therefore, BZDs may worsen PD symptoms [8]. Furthermore, animal studies have shown that BZDs could be a risk factor for pneumonia probably through the direct suppression of innate immunity [9]. Several studies have reported increased susceptibility to spontaneous bacterial infection and mortality in relation to the use of BZDs and BZRDs in an infection setting [9,10]. Most studies have shown that BZDs use is associated with an increased pneumonia risk [11,12]. Conversely, a population-based case-control study involving older adult people did not find a statistically significant association between BZDs and pneumonia [13]. The precise mechanism through which BZDs and BZRDs increase pneumonia risk are unknown.

However, few studies have examined how pneumonia risk is associated with the use of BZDs and BZRDs in older adult patients with PD. However, whether BZDs and BZRDs are associated with an increased pneumonia risk is still debatable. Our study hypothesized that the risk of pneumonia increases with the use of BZDs and BZRDs among patients with PD. The action mechanism underlying the development of pneumonia may differ between BZDs and BZRDs, and a drug-by-drug evaluation of such a mechanism is necessary. Therefore, in this study, we investigated pneumonia risk associated with the use of individual BZD and BZRD in patients with PD. Furthermore, we investigated the related risk factors for pneumonia by using nationwide data from Taiwan’s National Health Insurance (NHI) Research Database.

## 2. Materials and Methods

### 2.1. Database

This study conducted a secondary data analysis on the Longitudinal Health Insurance Database (LHID), which covers the period of 2001 to 2018 and is published by the Health and Welfare Data Science Center, Ministry of Health and Welfare (HWDC, MOHW). The LHID includes details of beneficiaries enrolled in Taiwan’s NHI program that covers up to 99% of its citizens. Hence, the LHID is a nationally representative health database for Taiwan. Information provided in the LHID, including detailed clinical records on outpatient visits, hospitalizations, diagnostic codes, and prescriptions, is highly concordant between NHI claims records and patients’ self-reports. Therefore, the LHID is frequently used to determine drug safety, including that relating to drug-induced pneumonia. The LHID is anonymous, and the HWDC deidentifies insured patients to protect their privacy. The requirement for informed consent was waived. This study protocol was approved by the Central Regional Research Ethics Committee of China Medical University, Taiwan (No. CRREC-109-011).

### 2.2. Study Participants

From the LHID, we identified patients aged ≥ 65 years with a diagnosis of PD (International Classification of Diseases, Ninth Revision, Clinical Modification [ICD-9-CM] 332 and International Classification of Diseases, Tenth Revision, Clinical Modification [ICD-10-CM] G20) at any time between 2002 and 2018. Older adult patients with a principal diagnosis of pneumonia (ICD-9-CM 480-486 and ICD-10-CM J12-J18) were included in the case group. To minimize potential confounding caused by unbalanced covariates in nonexperimental settings, we performed propensity score matching at a 1:4 ratio and included older adult patients without pneumonia as controls. The propensity score of the study was the probability that a patient received BZDs or BZRDs, calculated based on sex, age, income level, urbanization, and Charlson comorbidity index (CCI). After matching, a total of 551,975 older adult patients with PD were enrolled in the study between 2001 and 2018 in Taiwan.

### 2.3. Study Design

This was a case–control study designed to investigate pneumonia risk associated with the use BZDs and BZRDs drugs in older adult patients with PD. The dependent variable was the incident pneumonia and the independent variable was the use of individual BZD and BZRD. A patient was defined as using BZDs or BZRDs if they used any of the following, according to the Anatomic Therapeutic Chemical (ATC) classification system: BZDs, namely midazolam (N05CD08), triazolam (N05CD05), alprazolam (N05BA12), lorazepam (N05BA06), flunitrazepam (N05CD03), estazolam (N05CD04), oxazepam (N05BA04), diazepam (N05BA01), clonazepam (N03AE01), chlordiazepoxide (N05BA02), and flurazepam (N05CD01), and BZRDs, namely zolpidem (N05CF02) and zopiclone (N05CF01).

Patients’ medication using a year before pneumonia diagnosis was assessed. Exposure to BZDs and BZRDs was classified as “current” when the most recent prescription was within 30 days before pneumonia diagnosis. Furthermore, exposure to BZDs and BZRDs was classified as “recent” and “past” when prescriptions were 31 to 90 days and ≥90 days before pneumonia diagnosis, respectively. In addition, patients who were never prescribed BZDs and BZRDs before pneumonia diagnosis were included in the reference group.

The control variables in this study included sex, age, income level, urbanization, CCI score, and comorbidities related to pneumonia. The comorbidities were diabetes mellitus (ICD-9-CM 250 and ICD-10-CM E08-E13), hypertension (ICD-9-CM 401-405 and ICD-10-CM I10-I13 and I15), cerebrovascular disease (ICD-9 CM 430-438 and ICD-10-CM I60-I69), arrhythmia (ICD-9-CM 427 and ICD-10-CM I47-I49), upper respiratory tract infection (ICD-9-CM 465.9 and ICD-10-CM J00-06 and J30-39), heart failure (ICD-9-CM 428.0 and ICD-10-CM I50), asthma (ICD-9-CM 493 and ICD-10-CM J45), chronic obstructive pulmonary disease (COPD; ICD-9-CM 490-492 and 494-496 and ICD-10-CM J40-J44 and J47), periodontitis (ICD-9-CM 523 and ICD-10-CM K05.4), chronic kidney disease (ICD-9-CM 585 and ICD-10-CM N18), chronic liver disease (ICD-9-CM 571 and ICD-10-CM K70-K76), alcoholism (ICD-9-CM 303 and ICD-10-CM F10.2), Alzheimer disease (ICD-9-CM 331.0 and 290.1 and ICD-10-CM G30 and F00), rheumatoid arthritis (ICD-9-CM 714 and ICD-10-CM M05-M06 and M45), cancer (ICD-9-CM 140-239 and ICD-10-CM C00-C97), epilepsy (ICD-9-CM 345 and ICD-10-CM G40-G41), schizophrenia (ICD-9-CM 295-295.65 and 295.8-295.95 and ICD-10-CM F20-F20.9), bipolar disorder (ICD-9-CM 296.7 and ICD-10-CM F31.9), major depressive disorder (MDD; ICD-9-CM 296.3 and ICD-10-CM F32.9), and anxiety (ICD-9-CM 300.0 and ICD-10-CM F40 and F41).

### 2.4. Statistical Analysis

Descriptive statistics were first used to show distributions of participants’ characteristics, including sex, age, income level, urbanization, CCI score, and comorbidities related to pneumonia. We used the Chi-square test to examine the variables’ proportion because all variables were categorical data. We investigated the association between BZDs or BZRDs and pneumonia through a conditional logistic regression after adjusting all control variables. Statistical analysis was performed using SAS software version 9.4 (SAS Institute Inc., Cary, NC, USA). Statistical significance was indicated if *p*-value < 0.05.

## 3. Results

### 3.1. Baseline Characteristics

Table 1 presents the baseline characteristics of the study participants. After matching, 551,975 older adult patients with PD were included in the study. Among them, 110,395 and 441,580 patients received and did not receive a diagnosis of pneumonia, respectively. The age of patients with pneumonia was 80.14 ± 5.85 years. As expected, the distribution of sex, age, income level, urbanization, and CCI between the case and control groups were not significantly different after matching. The case group had patients with diabetes mellitus (31.06%), hypertension (61.42%), cerebrovascular disease (45.11%), arrhythmia (14.83%), upper respiratory tract infection (37.98%), congestive heart failure (14.44%), asthma (13.37%), COPD (38.75%), periodontitis (1.23%), chronic kidney disease (2.41%), chronic liver disease (8.28%), alcoholism (0.09%), Alzheimer’s disease (11.13%), rheumatoid arthritis (1.43%), cancer (14.40%), epilepsy (5.42%), schizophrenia (1.58%), bipolar disorder (1.42%), MDD (4.61%), or anxiety (13.61%).

### 3.2. Incidence Rate of Pneumonia with BZD Use

Table 2 presents the incidence rate of pneumonia with BZD use. Incident pneumonia was noted in 20.00%, 22.21%, 19.66%, and 20.44% of nonusers, current users, recent users, and past users of BZDs, respectively (*p* < 0.001). Compared with patients not receiving BZDs, current (adjusted odds ratio (aOR) = 1.25, 95% CI = 1.23 to 1.27) and past (aOR = 1.13, 95% CI = 1.11 to 1.15) users had a significantly higher incident pneumonia risk, whereas recent users had a non-significantly higher incident pneumonia risk (aOR = 1.01, 95% CI = 1.00 to 1.03).

With regard to individual BZDs, high incident pneumonia risk was observed in current, recent, and past users of midazolam (aOR = 3.93, 95% CI = 3.71 to 4.15; aOR = 1.26, 95% CI = 1.18 to 1.35; and aOR = 1.29, 95% CI = 1.26 to 1.32, respectively), lorazepam (aOR = 1.27, 95% CI = 1.24 to 1.31; aOR = 1.08, 95% CI = 1.05 to 1.11; and aOR = 1.16, 95% CI = 1.14 to 1.18, respectively), flunitrazepam (aOR = 1.15, 95% CI = 1.08 to 1.23; aOR = 1.11, 95% CI = 1.05 to 1.18; and aOR = 1.12, 95% CI = 1.08 to 1.15, respectively), estazolam (aOR = 1.28, 95% CI = 1.24 to 1.32; aOR = 1.14, 95% CI = 1.11 to 1.18; and aOR = 1.11, 95% CI = 1.10 to 1.13, respectively), and clonazepam (aOR = 1.10, 95% CI = 1.07 to 1.13; aOR = 1.05, 95% CI = 1.03 to 1.07; and aOR = 1.05, 95% CI = 1.03 to 1.06, respectively). Diazepam current users had a high incident pneumonia risk (aOR = 1.08, 95% CI = 1.03 to 1.13), whereas recent (aOR = 0.82, 95% CI = 0.79 to 0.85) and past (aOR = 0.90, 95% CI = 0.88 to 0.91) users had a low incident pneumonia risk. Low incident pneumonia risk was observed in current, recent, and past users of alprazolam (aOR = 0.94, 95% CI = 0.91 to 0.96; aOR = 0.88, 95% CI = 0.86 to 0.90; and aOR = 0.94, 95% CI = 0.93–0.95, respectively) and chlordiazepoxide (aOR = 0.75, 95% CI = 0.63 to 0.89; aOR = 0.70, 95% CI = 0.60 to 0.81; and aOR = 0.90, 95% CI = 0.87 to 0.94, respectively). Furthermore, incident pneumonia risk was low in current (aOR = 0.68, 95% CI = 0.56 to 0.83) and recent (aOR = 0.80, 95% CI = 0.68 to 0.93) users of oxazepam.

### 3.3. Incidence Rate of Pneumonia with BZRD Use

Table 3 presents the incidence rate of pneumonia with BZRD use. Incident pneumonia was noted in 20.05%, 19.44%, 18.58%, and 21.28% of nonusers, current users, recent users, and past users of BZRDs, respectively (*p* < 0.001). Compared with patients who did not receive BZRDs, recent users had a high incident pneumonia risk (aOR = 1.08, 95% CI = 1.06 to 1.11), whereas past users had a low incident pneumonia risk (aOR = 0.89, 95% CI = 0.88 to 0.91).

Among BZRDs, current (aOR = 0.94, 95% CI = 0.91 to 0.97) and recent (aOR = 0.86, 95% CI = 0.86 to 0.91) users of zolpidem had a low risk of incident pneumonia, whereas past users of the drug had a high risk of incident pneumonia (aOR = 1.07, 95% CI = 1.06 to 1.09). Furthermore, current (aOR = 1.14, 95% CI = 1.08 to 1.20), recent (aOR = 1.07, 95% CI = 1.02 to 1.11), and past (aOR = 1.11, 95% CI = 1.08 to 1.13) users of zopiclone had a high risk of incident pneumonia.

## 4. Discussion

Different drugs have different pharmaceutical properties that can substantially affect biological properties. These individual BZD or BZRD drugs may have different action mechanisms that drive the development of pneumonia. This case–control study analyzed data of 551,975 older adult patients with PD between 2001 and 2018 in Taiwan. Our study revealed that for individual BZDs, midazolam, lorazepam, flunitrazepam, estazolam, and clonazepam were associated with increased incident pneumonia risk in current, recent, and past users in elderly patients with PD. However, several individual BZDs such as alprazolam and chlordiazepoxide were associated with decreased incident pneumonia risk in current, recent, and past users. Among BZRDs, zolpidem current and recent users had a low incident pneumonia risk, whereas past users had a high incident pneumonia risk. Furthermore, zopiclone current, recent, and past users had a high incident pneumonia risk.

For treating sleep disturbance among patients with PD, several sedative-hypnotics are prescribed, including BZDs and BZRDs, which are mainstay treatments for insomnia [5]. BZDs, as GABA modulators, are commonly used for the treatment of sleep disorders, anxiety, and some forms of depression [14]. PD is the second most common age-related motoric neurodegenerative disorder, which is likely to lead to oropharyngeal dysphagia and may increase aspiration pneumonia risk [2]. Several studies have indicated that BZDs and BZRDs are associated with an increased pneumonia risk in older adult patients [11,12]. However, a study in older adult patients did not find a statistically significant association between BZDs and pneumonia (OR = 1.08, 95% CI = 0.84 to 1.47), which may partly be due to low numbers of patients exposed to BZDs in this study [13]. By contrast, another study showed that BZDs may be associated with a decreased pneumonia risk. However, this study used questionnaires to determine drug exposure and thus has potential recall and reporting bias [15]. Previous studies have not specifically examined the association between BZRAs and pneumonia, and the small sample size may have decreased the power of the study [15]. The association of the use of BZDs and BZRDs with pneumonia risk has received increasing attention but is still controversial. However, no study has explored whether patients with PD who use BZDs and BZRDs have increased pneumonia risk. Therefore, our study was conducted to investigate pneumonia risk associated with the use of BZDs and BZRDs among older adult patients with PD in the Taiwan population. The large sample size used was representative of the population and allowed for robust findings in our analysis. To the authors’ knowledge, this is the first study to directly evaluate the association of BZDs and BZRDs with pneumonia risk among older adult patients with PD.

Pneumonia risk among older adult patients with PD varies depending on the use of BZDs or BZRDs. As for individual BZDs, midazolam, lorazepam, flunitrazepam, estazolam, and clonazepam were associated with increased incident pneumonia risk in current, recent, and past users. However, several BZDs such as alprazolam and chlordiazepoxide were associated with decreased incident pneumonia risk in current, recent, and past users. Among BZRDs, zolpidem current and recent users had a low incident pneumonia risk, whereas past users had a high incident pneumonia risk. Furthermore, current, recent, and past users of zopiclone had a high incident pneumonia risk.

In our study, compared with patients not receiving BZDs, current and past users of BZDs had a significantly increased incident pneumonia risk. Several mechanisms have been proposed in animal and physiological studies to explain the possible association of BZD or BZRD use with pneumonia risk. First, BZDs can sedate, may prolong hypoventilation duration [16], and may lead to pneumonia with increased aspiration risk. The clearing of oral salivary secretions may be impaired during deep sedation, particularly during peak drug concentrations. The swallowing of saliva is strongly inhibited during deep sleep [17]. Second, animal studies have indicated that GABA agonists can decrease extracellular striatal dopamine concentrations, and, therefore, BZDs may worsen PD symptoms [8]. Third, according to the anticholinergic burden score for drugs in Germany, BZDs and BZRDs have weak anticholinergic effects [18]. Medications exerting an anticholinergic effect may lead to oropharyngeal swallowing impairment, which results in aspiration pneumonia [19]. Anticholinergic drugs are one of the risk factors for pneumonia in older adult patients. As BZDs and BZRDs have weak anticholinergic effects, they may be associated with pneumonia risk among older adult patients with PD. Fourth, BZDs relax the lower esophageal sphincter and increase reflux events during sleep [20], which could increase pneumonia risk. BZDs decrease lower esophageal sphincter pressure, perhaps through the activation of peripherally situated GABA receptors [21]. Fifth, BZDs may directly influence the pulmonary system by activating GABA receptors located in the peripheral nervous system or peripheral tissue [21]. Importantly, mouse and human immune cells, (including alveolar macrophage) express GABA receptors [9], thus serving as translational evidence that humans may be at risk. BZDs depress central respiratory drive and decrease inspiratory and expiratory respiratory muscle strength in a dose-dependent manner, thus reducing respiration [22]. Similarly, BZRDs may cause respiratory depression by decreasing respiratory muscle strength, suppressing central respiratory drive, and increasing upper airway resistance [23]. Finally, BZRAs also suppress peripheral immunity through the activation of GABA receptors [9] or peripheral BZD receptors (PBRs) [24]. Several studies have indicated that the activation of GABA receptors may weaken the immune system [9]. An in vivo study showed that PBR ligands inhibit both inflammatory cytokine production in acute inflammation [25] and macrophage production of several key immune response cytokines [26].

Human epidemiological data have revealed that BZD use is a risk factor for complicated community-acquired lower respiratory tract infection [27]. BZDs are associated with increased pneumonia risk because BZDs have a relatively high affinity for both intracellular and cell surface receptors, whereas the GABAergic mechanism is probably responsible for BZRD-induced pneumonia owing to the lower affinity of BZRD for PBRs [28]. Different drugs have different pharmaceutical properties that can substantially affect biological properties. These individual BZD or BZRD drugs may have different action mechanisms that drive the development of pneumonia. To investigate the immune response among older adult patients with PD for each BZD or BZRD is necessary.

Our study revealed that several BZDs increase incident pneumonia risk, including midazolam, lorazepam, clonazepam, flunitrazepam, and estazolam. Midazolam use was likely to result in the development of pneumonia among older PD patients receiving BZDs, and midazolam users had a high incident pneumonia risk. Some possible mechanisms have been suggested that support the relationship between midazolam use and pneumonia risk. Midazolam probably acts on PBR, impairing the response to infection in mice, mainly through the inhibition of macrophage spread, phagocytosis function, and oxidative bursts of neutrophils and macrophages [11]. Another possible mechanism is that midazolam significantly increases the incidence of pharyngeal dysfunction from 16% to approximately 48% [29]. A study showed that at 2 h after midazolam administration, the swallowing reflex was depressed, thus increasing the latency time to initiate a swallowing action even after recovery to normal consciousness [30]. Our study found that lorazepam users had a high incident pneumonia risk. We found that clonazepam users had a high incident pneumonia risk. A study indicated that compared with other BZDs, clonazepam has a strong binding capacity to PBRs in rat aortic smooth muscles [31] and can thus impair the response to infection.

In theory, BZDs can normalize these brain areas in patients with hypoactive GABA [32]. In general, anxiogenic BZDs suppress the immune response, whereas anxiolytic BZDs may protect the individual from stress-induced immunosuppression [33]. Our study revealed that several BZDs decreased incident pneumonia risk, including alprazolam, oxazepam, and chlordiazepoxide. Alprazolam users had a low risk of incident pneumonia. An in vitro study demonstrated that triazolo-BZDs (alprazolam and triazolam) do not modify the phagocytosis and killing by human polymorphonuclear cells and monocytes. Alprazolam was found to be efficacious in controlling PD symptoms [34]. Our study revealed that chlordiazepoxide users had a low incident pneumonia risk. This may be because chlordiazepoxide is a BZD with anxiolytic and sedative-hypnotic properties [32]. However, the mechanism remains unclear, and further investigation is necessary. Furthermore, our study revealed that diazepam current users had a high incident pneumonia risk, whereas recent and past users had a low incident pneumonia risk. Another study reported that the dose-dependent effect of diazepine (ranging from stimulation to inhibition) may be caused by different BZD receptors involved in the process [35]. Previous studies have suggested that the long-term use of BZDs may weaken the immune system, although no conclusive evidence is available to support this claim [36].

BZRDs exhibit greater selectivity than BZDs do for GABA receptors containing alpha1 subunits, which exert considerable hypnotic effects [37]. BZD-induced pneumonia has a relatively high affinity for PBRs, whereas BZRD-induced pneumonia has a low affinity for PBRs [28]. Our study revealed that the risk of BZRD-induced pneumonia is lower than that of BZD-induced pneumonia among patients with PD. Furthermore, we found that compared with patients not receiving BZRDs (zolpidem and zopiclone), recent BZRD users had an increased incident pneumonia risk, whereas past users had a decreased incident pneumonia risk.

We observed that current and recent users of Zolpidem had a low incident pneumonia risk, whereas past users had a high incident pneumonia risk. Some possible mechanisms underlie the relationship between zolpidem use and pneumonia risk. Zolpidem may increase sleep apnea incidence and suppress the respiratory drive [38]. A study observed that zolpidem use increased infection risk in patients with sleep disturbance [39]. Several studies have shown that zolpidem can improve neuropsychiatric symptoms and motor dysfunction in patients with PD [40,41,42]. We found that zopiclone users had a high risk of incident pneumonia. Zopiclone may adversely affect the immune system, increasing the risk of infections. Obiora et al. found an approximately two-fold increase in pneumonia risk with BZD or zopiclone use within 30 days of therapy [43]. However, this study was limited by the effects of BZD and BZRD use not being distinguished. Furthermore, the effect of zopiclone on the immune system in patients with PD is unknown.

This study has several strengths. First, we used a large sample size that was representative of the entire Taiwanese population, which allowed for robust findings. This is a nationwide population-based case–control study with nearly complete follow-up information with regard to health care institutes among the whole study population, and the data set is routinely monitored for diagnostic accuracy by the NHI Bureau of Taiwan. Second, the follow-up period of this study was divided into current use (<30 days), recent use (31–90 days), and past use (>90 days) to investigate the relationship between drug use and pneumonia risk. Thus, the relationship between the drug and pneumonia risk at different stages of its use could be studied.

This study has several limitations that must be addressed. First, some factors related to pneumonia cannot be obtained from the LHID, such as alcohol consumption and smoking status, chest X-ray results, pneumonia etiology, and laboratory parameters. The LHID only can present information that are part of a health insurance declaration, and medical information in uninsured treatments cannot be obtained. Thus, the use of BZDs or BZRDs may be underestimated. Third, inclusion of prevalent BZD and BZRD users could potentially result in an underestimation of the overall risks because they might have developed a tolerance for pneumonia. Fourth, the study used only the ICD code to define diseases and did not consider medical procedure codes. Hence, pneumonia may be overrepresented. Fifth, this study was an observational study, which precluded any inference that BZD or BZRD use causes pneumonia. In future studies, information from other relational databases or questionnaires must be obtained to infer causality.

## 5. Conclusions

Older patients with PD receiving BZDs and BZRDs had associated with the risk of pneumonia. Among these medications, BZDs, such as midazolam, lorazepam, flunitrazepam, estazolam, and clonazepam, had the highest risk of pneumonia. Regarding the use of BZRDs, zopiclone also increased incident pneumonia risk. Clinicians should pay attention to the risk of pneumonia in older patients with PD who receive BZDs and BZRDs.

## Figures and Tables

**Table 1:** Baseline characteristics of older adult patients with Parkinson’s disease.

Variables	Pneumonia				*p*-Value
	Without		With		
	N	%	N	%	
Total	441,580	100.00	110,395	100.00	
Gender					0.005
Female	220,311	49.89	54,554	49.42	
Male	221,269	50.11	55,841	50.58	
Age (year)					0.023
65–70	16,185	3.67	4064	3.68	
70–75	64,198	14.54	16,039	14.53	
75–80	119,984	27.17	30,080	27.25	
80–85	136,585	30.93	34,545	31.29	
≥85	104,628	23.69	25,667	23.25	
Mean ± SD	79.94 ± 5.74		80.14 ± 5.85		
Income level					0.965
Low income	235,250	53.27	58,886	53.34	
Middle income	95,549	21.64	23,860	21.61	
High income	110,781	25.09	27,649	25.04	
Urbanization					0.463
Level 1	100,034	22.65	25,063	22.70	
Level 2	126,121	28.56	31,408	28.45	
Level 3	64,910	14.70	16,344	14.81	
Level 4	80,281	18.18	19,887	18.01	
Level 5	17,518	3.97	4321	3.91	
Level 6	29,281	6.63	7462	6.76	
Level 7	23,435	5.31	5910	5.35	
CCI score					0.992
0	10,504	2.38	2629	2.38	
1	45,358	10.27	11,340	10.27	
2	85,582	19.38	21,350	19.34	
≥3	300,136	67.97	75,076	68.01	
Diabetes mellitus					<0.001
No	291,857	66.09	76,109	68.94	
Yes	149,723	33.91	34,286	31.06	
Hypertension					<0.001
No	167,083	37.84	42,593	38.58	
Yes	274,497	62.16	67,802	61.42	
Cerebrovascular disease					<0.001
No	266,852	60.43	60,600	54.89	
Yes	174,728	39.57	49,795	45.11	
Arrhythmia					<0.001
No	381,796	86.46	94,022	85.17	
Yes	59,784	13.54	16,373	14.83	
Upper respiratory tract infection					<0.001
No	269,133	60.95	68,469	62.02	
Yes	172,447	39.05	41,926	37.98	
Congestive heart failure					<0.001
No	388,929	88.08	94,455	85.56	
Yes	52,651	11.92	15,940	14.44	
Asthma					<0.001
No	401,943	91.02	95,638	86.63	
Yes	39,637	8.98	14,757	13.37	
COPD					<0.001
No	335,009	75.87	67,621	61.25	
Yes	106,571	24.13	42,774	38.75	
Periodontitis					<0.001
No	434,730	98.45	109,035	98.77	
Yes	6850	1.55	1360	1.23	
Chronic kidney disease					0.544
No	430,789	97.56	107,732	97.59	
Yes	10,791	2.44	2663	2.41	
Chronic liver disease					<0.001
No	396,726	89.84	101,256	91.72	
Yes	44,854	10.16	9139	8.28	
Alcoholism					0.713
No	441,204	99.91	110,297	99.91	
Yes	376	0.09	98	0.09	
Alzheimer disease					<0.001
No	408,047	92.41	98,103	88.87	
Yes	33,533	7.59	12,292	11.13	
Rheumatoid arthritis					<0.001
No	433,895	98.26	108,813	98.57	
Yes	7685	1.74	1582	1.43	
Cancer					<0.001
No	369,884	83.76	94,499	85.60	
Yes	71,696	16.24	15,896	14.40	
Epilepsy					<0.001
No	431,457	97.71	104,411	94.58	
Yes	10,123	2.29	5984	5.42	
Schizophrenia					<0.001
No	438,195	99.23	108,656	98.42	
Yes	3385	0.77	1739	1.58	
Bipolar disorder					<0.001
No	436,522	98.85	108,831	98.58	
Yes	5058	1.15	1564	1.42	
Major depressive disorder					<0.001
No	420,971	95.33	105,309	95.39	
Yes	20,609	4.67	5086	4.61	
Anxiety					<0.001
No	363,846	82.40	95,365	86.39	
Yes	77,734	17.60	15,030	13.61	

**Table 2:** Pneumonia risk associated with benzodiazepines use.

Variables	Pneumonia								
	Without		With		*p*-Value ^1^	Adjusted Model ^2^			
	N	%	N	%		OR	95% CI		*p*-Value
Any one of BZD									
No	339,345	80.69	81,203	19.31		1			
Current users	102,235	77.79	29,192	22.21	<0.001	1.25	1.23	1.27	<0.001
Recent users	145,250	80.34	35,553	19.66	<0.001	1.01	1.00	1.03	0.093
Past users	324,647	79.56	83,397	20.44	<0.001	1.13	1.11	1.1	<0.001
Short-acting									
Midazolam									
No	438,869	80.30	107,694	19.70		1			
Current users	2711	50.09	2701	49.91	<0.001	3.93	3.71	4.15	<0.001
Recent users	4033	75.54	1306	24.46	<0.001	1.26	1.18	1.35	<0.001
Past users	28,624	74.02	10,048	25.98	<0.001	1.29	1.26	1.32	<0.001
Triazolam									
No	439,377	80.00	109,828	20.00		1			
Current users	2203	79.53	567	20.47	0.536	1.05	0.95	1.15	0.324
Recent users	3074	81.82	683	18.18	0.005	0.93	0.86	1.01	0.101
Past users	15,204	78.87	4073	21.13	<0.001	1.00	0.96	1.04	0.952
Intermediate-acting									
Alprazolam									
No	412,901	79.88	104,009	20.12		1			
Current users	28,679	81.79	6386	18.21	<0.001	0.94	0.91	0.96	<0.001
Recent users	42,468	82.90	8758	17.10	<0.001	0.88	0.86	0.90	<0.001
Past users	161,774	80.48	39,232	19.52	<0.001	0.94	0.93	0.95	<0.001
Lorazepam									
No	417,686	80.23	102,900	19.77		1			
Current users	23,894	76.12	7495	23.88	<0.001	1.27	1.24	1.31	<0.001
Recent users	35,122	79.26	9189	20.74	<0.001	1.08	1.05	1.11	<0.001
Past users	162,821	78.40	44,867	21.60	<0.001	1.16	1.14	1.18	<0.001
Flunitrazepam									
No	437,602	80.03	109,214	19.97		1			
Current users	3978	77.11	1181	22.89	<0.001	1.15	1.08	1.23	<0.001
Recent users	5355	78.38	1477	21.62	<0.001	1.11	1.05	1.18	<0.001
Past users	19,118	76.64	5827	23.36	<0.001	1.12	1.08	1.15	<0.001
Estazolam									
No	423,084	80.18	104,590	19.82		1			
Current users	18,496	76.11	5805	23.89	<0.001	1.28	1.24	1.32	<0.001
Recent users	27,496	78.44	7558	21.56	<0.001	1.14	1.11	1.18	<0.001
Past users	98,439	78.22	27,416	21.78	<0.001	1.11	1.10	1.13	<0.001
Oxazepam									
No	440,855	79.99	110,269	20.01		1			
Current users	725	85.19	126	14.81	<0.001	0.68	0.56	0.83	<0.001
Recent users	1009	83.80	195	16.20	<0.001	0.80	0.68	0.93	0.005
Past users	7257	80.17	1,795	19.83	0.683	0.96	0.91	1.01	0.106
Long-acting									
Diazepam									
No	431,029	80.02	107,603	19.98		1			
Current users	10,551	79.08	2792	20.92	0.007	1.08	1.03	1.13	0.001
Recent users	16,796	83.65	3284	16.35	<0.001	0.82	0.79	0.85	<0.001
Past users	136,677	80.76	32,570	19.24	<0.001	0.90	0.88	0.91	<0.001
Clonazepam									
No	409,759	80.14	101,522	19.86		1			
Current users	31,821	78.20	8873	21.80	<0.001	1.10	1.07	1.13	<0.001
Recent users	47,504	79.45	12,286	20.55	<0.001	1.05	1.03	1.07	<0.001
Past users	146,600	79.23	38,436	20.77	<0.001	1.05	1.03	1.06	<0.001
Chlordiazepoxide									
No	440,728	79.99	110,244	20.01		1			
Current users	852	84.95	151	15.05	<0.001	0.75	0.63	0.89	0.001
Recent users	1311	86.48	205	13.52	<0.001	0.70	0.60	0.81	<0.001
Past users	14,166	81.76	3161	18.24	<0.001	0.90	0.87	0.94	<0.001
Flurazepam									
No	440,857	80.01	110,164	19.99		1			
Current users	723	75.79	231	24.21	0.001	1.19	1.02	1.39	0.024
Recent users	1057	78.47	290	21.53	0.160	1.10	0.97	1.26	0.146
Past users	7826	79.77	1985	20.23	0.562	0.93	0.89	0.98	0.008

^1^ Chi-square test. ^2^ All models were analyzed via the conditional logistic regression. Extraneous factors adjusted in the model contained all comorbidities.

**Table 3:** Pneumonia risk associated with benzodiazepine related drugs use.

Variables	Pneumonia							
	Without		With		*p*-Value ^1^	Adjusted Model ^2^		
	N	%	N	%		OR	95% CI	*p*-Value
Any one of BZRD								
No	407,779	79.95	102,238	20.05		1		
Current users	33,801	80.56	8157	19.44	0.003	1.02	0.99–1.04	0.239
Recent users	47,714	81.42	10,891	18.58	<0.001	1.08	1.06–1.11	<0.001
Past users	158,308	78.72	42,787	21.28	<0.001	0.89	0.88–0.91	<0.001
Zolpidem								
No	414,818	79.91	104,319	20.09		1		
Current users	26,762	81.50	6076	18.50	<0.001	0.94	0.91–0.97	<0.001
Recent users	38,005	82.28	8187	17.72	<0.001	0.88	0.86–0.91	<0.001
Past users	140,526	78.99	37,379	21.01	<0.001	1.07	1.06–1.09	<0.001
Zopiclone								
No	433,958	80.05	108,153	19.95		1		
Current users	7622	77.27	2242	22.73	<0.001	1.14	1.08–1.20	<0.001
Recent users	11,014	78.50	3017	21.50	<0.001	1.07	1.02–1.11	0.003
Past users	50,767	77.49	14,744	22.51	<0.001	1.11	1.08–1.13	<0.001

^1^ Chi-square test. ^2^ All models were analyzed via the conditional logistic regression. Extraneous factors adjusted in the model contained all comorbidities.

## Data Availability

The National Health Insurance Database used to support the findings of this study were provided by the Health and Welfare Data Science Center, Ministry of Health and Welfare (HWDC, MOHW) under license and so cannot be made freely available. Requests for access to these data should be made to HWDC (https://dep.mohw.gov.tw/dos/np-2497-113.html, accessed on 12 July 2021).
